# Histological and Radiological Analyses of a Maxillary Sinus Lift with Extensive Drilling of the Schneider Membrane Using Xenogeneic Bone

**DOI:** 10.1155/2014/898031

**Published:** 2014-09-02

**Authors:** Marcelo M. Romano, Júlia A. Smanio, Lorraine B. Ferreira, Victor E. Arana-Chavez, Mário S. Soares

**Affiliations:** ^1^Integrated Clinic Discipline, Department of Stomatology, School of Dentistry, University of São Paulo, 05508-000 São Paulo, SP, Brazil; ^2^Department of Biomaterials and Oral Biology, School of Dentistry, University of São Paulo, 05508-000 São Paulo, SP, Brazil

## Abstract

The objective of this study is to report a clinical case of maxillary sinus with lyophilized, xenogeneic graft, in which, despite a large perforation of the sinus membrane, the surgery was not aborted and the results of histological examinations indicate bone neoformation in the surgical area. *Results*. This case showed that the biomaterials evaluated in this study and the procedure used to place them proved to be biocompatible and presented high osteogenic potential, leading to a successful surgery and osseointegration implant. *Conclusion*. Positioning Schneider's membrane and filling it with the graft biomaterial helped to achieve the desired osteoconduction and proliferation of bone cells even though the patient had a large perforation of the sinus membrane.

## 1. Introduction

The posterior maxillary region is difficult to rehabilitate with dental implants due to two main limiting factors: maxillary sinus pneumatization and low bone density [1]. One treatment currently used to obtain sufficient vertical bone height for implant placement is a sinus membrane lift [1].

Despite a success rate of 95 to 97%, perforation of the sinus membrane is a routine complication when implementing this technique, principally due to the presence of septa, anatomical accidents, and/or failures by the surgeon [2].

Several attempts have been made to classify membrane perforations. Fugazzotto and Vlassis [2] proposed five classes based on their location and difficulty to repair. According to this classification, small perforations are easily treated with maneuvers in the sinus membrane and the use of absorbable membranes and medium size perforations can be treated by absorbable or sutured membranes; however, in cases involving large perforations, the sinus lift surgery should be aborted and postponed until a later date [2].

Chen et al. [3] presented a system of classifications and reparations of sinus membrane perforations while performing sinus augmentation from the crestal approach. The classification consists of five classes of varying perforation severity and size, each with corresponding management techniques. Class 1 (<2 mm) and class 2 perforations (2–5 mm), when corrected, do not compromise the sinus lift surgery. However, according to this classification, perforations greater than 6 mm mean the patient is not eligible for a sinus lift procedure [4].

Materials that are to be implanted in the body must present specific characteristics, including biocompatibility, low allergenic and carcinogenic potential, and resistance to deformation and resorption [5]. The introduction of materials, such as collagen membranes and grafting biomaterials, is increasingly the subject of studies, since they possess biocompatibility, permit bone migration and surface colonization by osteogenic cells, and present greater capacity to mimic the physical properties of bone in relation to soft tissues, in comparison with other materials. The components produced by the dissociation of the material possess the capacity to integrate with the neoformed bone [6, 7].

The purpose of this study was to present a clinical case with lyophilized, xenogeneic graft, in which a large perforation of the sinus membrane occurred and show the results of histological examinations in the region of bone neoformation.

## 2. Clinical Case Report

A 62-year-old woman with leukoderma was attended at the outpatient clinic of the Center for Studies and Extension in Integrated Therapies (*Núcleo de Estudos e Extensão em Tratamentos Integrados*, NEXTI) of the University of São Paulo, School of Dentistry, regarding the rehabilitation of edentulous regions. She complained of dissatisfaction with the esthetic factors and difficulty when masticating. The patient's medical history included depression, which was being controlled with antidepressive drugs. Clinical and radiographic examination revealed the absence of elements 22, 26, 27, 36, and 37.

An initial assessment using cone-beam computed tomography with 1 mm thickness cross-sections of the region determined that insufficient bone height remained (2 mm) of elements 26 and 27 due to pneumatization of the right maxillary sinus, thus limiting treatment involving osseointegrated implants without prior bone reconstruction.

The initial treatment plan proposed performing a sinus lift in the region of tooth 26, filled with lyophilized xenogeneic bone graft (Bio-Oss, Geistlich Pharma AG, Wolhusen, Switzerland) and coated with collagen membrane (Bio-Guide, Geistlich Pharma AG, Wolhusen, Switzerland) to stabilize the graft.

**Figure 1:**
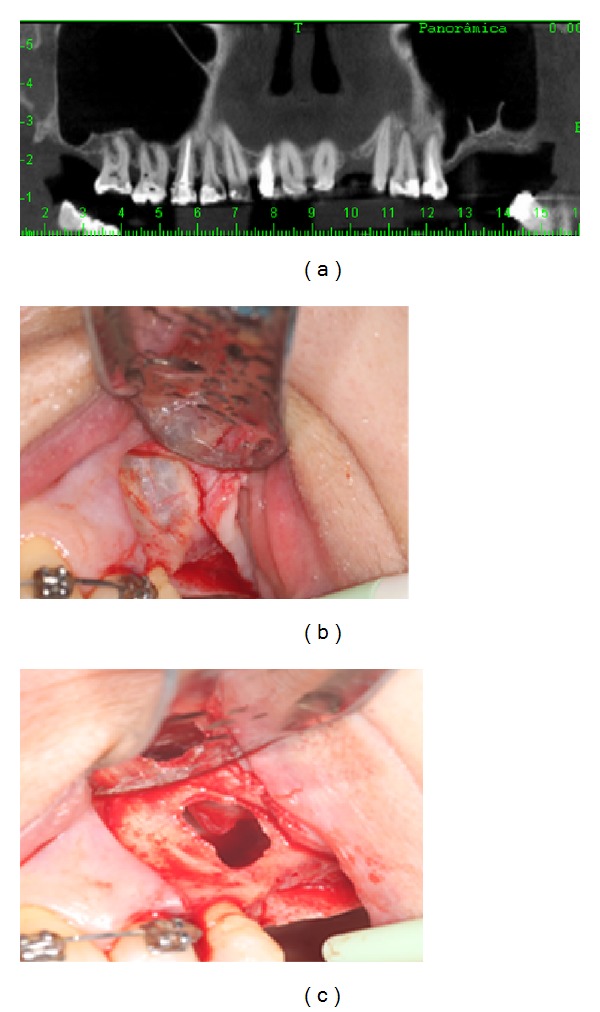
The initial computerized tomography (a), osteotomy of the lateral wall of the left maxillary sinus (b), and the sinus membrane rupture and subsequent lift (c).

The patient was medicated with 1 g of amoxicillin (2 tablets of 500 mg) one hour prior to the procedure. Extraoral antisepsis was performed using 2% chlorhexidine and intraoral with 0.12% chlorhexidine, followed by field block anesthesia and local infiltration with lidocaine hydrochloride and epinephrine 1 : 100,000. During surgery, a supracrestal incision was performed with a 15 C blade, discretely toward the palate, in the 26-27 region, and a relaxing incision mesial to 25, with detachment of a full thickness flap. Osteotomy of the lateral wall of the right maxillary sinus was performed with spherical carbide bur number 10 for armored straight piece, under copious irrigation with 0.9% saline. During the detachment of the sinus membrane with specific curettes, the presence of a large size perforation was observed (Figure 1).

**Figure 2:**
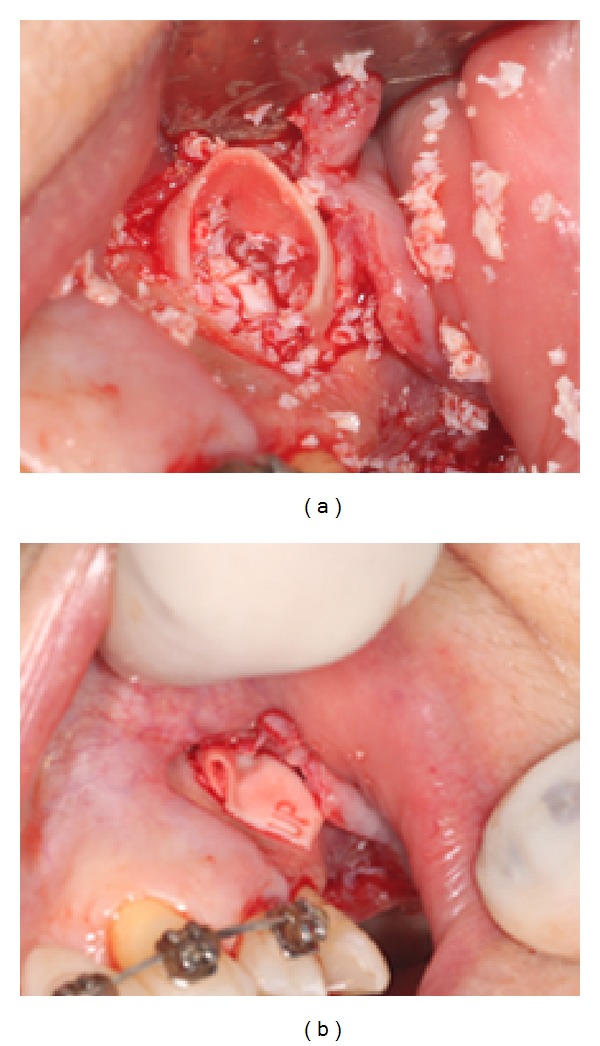
Filling the maxillary sinus with bone particles (a) and coating it with collagen membrane (b).

The sinus membrane was carefully lifted and, with the aid of resorbable collagen (Bio-Guide), the membrane perforation was protected for the purpose of containing the graft material (2 g flask of Bio-Oss) (Figure 2).

Next, the local flap was repositioned and sutured with 4.0 silk thread. The patient was prescribed 500 mg of amoxicillin, every 8 hours, for 7 days.

Six months later, the time required for maturation of the grafted tissue, a periapical radiograph, was taken of the site that determined that the maxillary sinus membrane lift had been successful.

**Figure 3:**
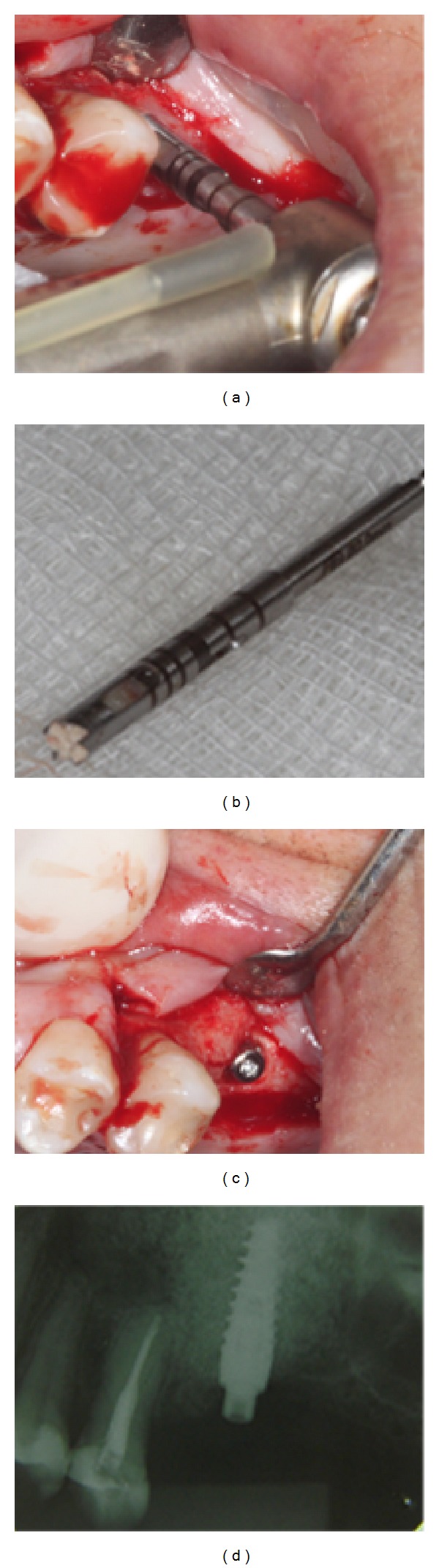
Removal of a fragment of bone tissue from the graft area (a) with a trephine number 2 bur following treatment (b). Implant placement (c) and final periapical radiograph (d).

During a second surgery, performed five months after the first, a fragment of bone tissue was removed with a trephine number 2 bur from in the grafted area and carefully identified. This bone was placed in a container with fixative solution for subsequent histological analysis of the bone tissue. A Drive CM implant (Neodent, Curitiba, PR, Brazil) measuring 3.5 mm in diameter and 11.5 mm in length was then installed, with a final insertion torque of 45 Ncm (Figure 3).

**Figure 4:**
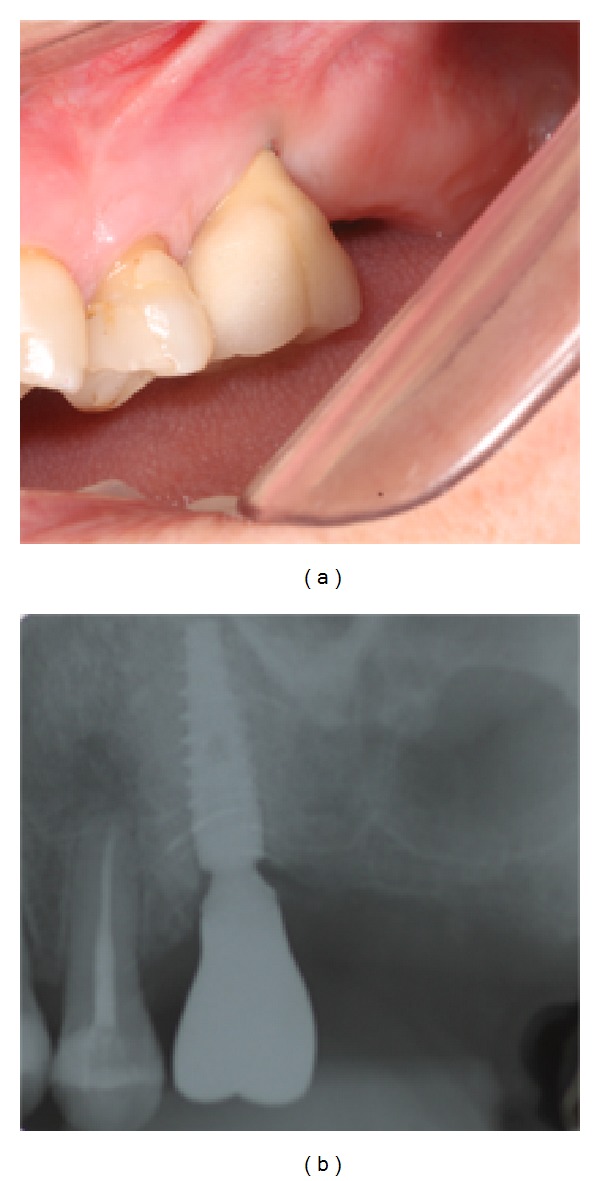
Finalized porcelain-fused-to-metal crown (a) and final X-ray (b).

The reopening and installation of healing abutments were performed following the six-month interval required for osseointegration of the implants. The prosthetic component was installed later and a mold was made for fabrication of the provisional and definitive porcelain-fused-to-metal (PFM) crowns (Figure 4).

### 2.1. Histological Processing

For the histological processing, the specimen was quickly immersed in a beaker containing 40 mL of fixative. The fixative consisted of 4% formaldehyde (prepared from paraformaldehyde) + 0.1% glutaraldehyde buffered with 0.1 M sodium cacodylate and pH 7.2. Next, the beaker containing the material and fixer was placed in a container with ice for subsequent microwave irradiation in a Pelco 3440 oven (Ted Pella, Redding, CA, USA) operating at 100% power for 3 cycles of 5 min each, at a maximum temperature of 37°C [4]. Following fixation in the microwave oven, the specimens were transferred to a fixative for 6 h at room temperature, after which the material was maintained overnight at 4°C. The following day, the specimen was washed in cacodylate buffer 0.01 M sodium, pH 7.2 and decalcified in 4.13% EDTA for 30 days. The sample was then dehydrated in serial, increasing concentrations of ethanol, clarified in xylene, infiltrated, and subsequently embedded in paraffin (Sigma, St. Louis, USA). Four mm thick sections were stained with hematoxylin and eosin and the images were analyzed and captured under an Olympus BX-60 light microscope coupled to the Cell F image capture and analysis system (Olympus, Tokyo, Japan).

## 3. Results

**Figure 5:**
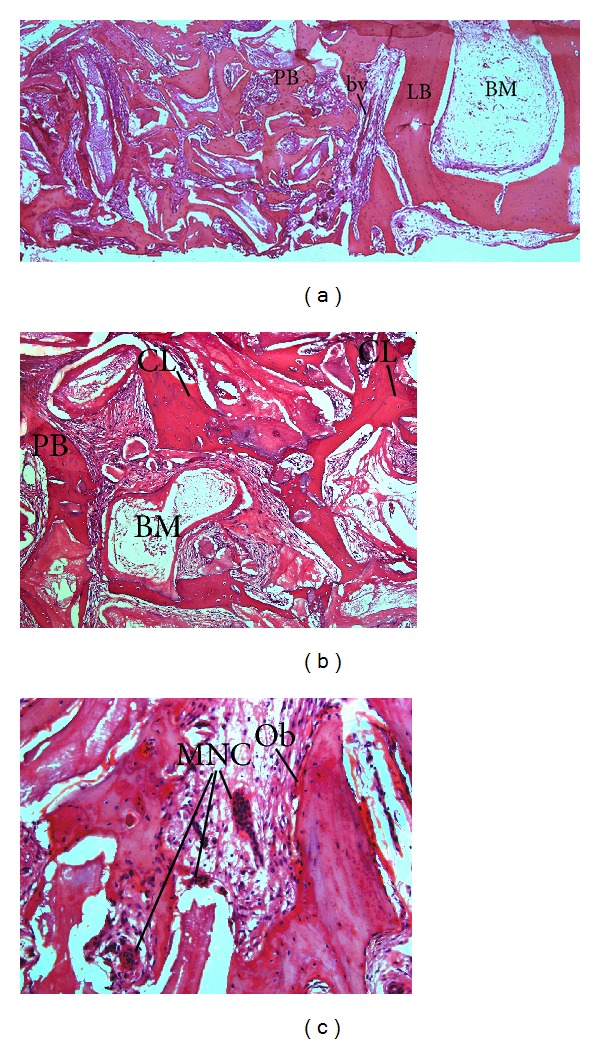
Light microscopy. The upper micrograph (a) illustrates a significant increase in bone neoformation. A layer of lamellar bone (LB) surrounding the biomaterial (BM) is present after 150 days of healing. No signs of inflammation were observed, but the presence of blood vessels (bv) is evident. In (b), the layer of neoformed bone (PB) and a cement line (cl) are present in the center of the remodeling region. At higher magnification, in the area adjacent to the lamellar bone (c), islets of newly formed bone can be observed, lined by a thin layer of osteoblasts (Ob). Multinuclear cells (MNC) are also observed between the growing trabecular bone towards the center of the bone defect. Hematoxylin and eosin staining. Original micrographs at (a) 40x, (b) 100x, and (c) 200x magnification.

The images obtained by histological analysis showed the prior occurrence of an intense bone tissue neoformation, together with the presence of blood vessels and capillaries between the newly formed matrix, with large quantities of osteocytes in the respective bone regions. In addition, in certain sections, bone with a lamellar appearance was observed adjacent to the recently deposited primary bone, which can be explained by the osteoconductive capacity of the biomaterial. This biomaterial maintained the region free from the invasion of connective tissue and provided a scaffold for cell proliferation and osteoblast differentiation. When properly stimulated by the biomaterial, active osteoblasts secreted the osteoid matrix located adjacent to the surfaces of mature bone matrix with a lamellar appearance. The biodegradation of the material was concomitant to the bone remodeling process. In this surgical period, we observed the presence of traces of degradation in the sections analyzed. The preexisting bone edges showed integration with the neoformed tissue and possibly the source of the osteoconductive potential of the biomaterial in the region of defective bone (Figure 5).

## 4. Discussion

The biological concepts of bone regeneration are used in studies that assess the osteogenic potential of biomaterials; however, the use of experimental models can only simulate conditions under which regeneration occurs to a limited extent [8, 9]. Based on this premise and seeking to consolidate such models with clinical results, this study evaluated the use of a biomaterial graft (2 g flask of Bio-Oss), adapted by the use of a resorbable collagen membrane, in a case in which extensive perforation of the sinus membrane occurred. According to the literature, membrane perforations larger than 10 mm render maxillary sinus lift surgery unfeasible [3].

A few studies have reported alternatives techniques for repairing large perforations. Pikos [10] proposed the use of a slow resorbing type I collagen membrane for repairing large and complete sinus membrane perforations. Despite the biocompatibility and semirigid structural integrity of this membrane, the need for external tack fixation demands more time and greater experience of the surgeon. Another technique describes the use of a pedicled buccal fat pad to close the large perforation; however, this technique can involve certain postoperative complications, including partial necrosis, fibrosis, shrinkage, retraction, and a variable degree of distortion [11]. To obliterate a large perforation, some researchers have sutured the sinus membrane to the bone directly lateral to the osteotomy site with a resorbable material [12]; this is a very delicate maneuver. In contrast, the case described in this study shows a simple technique that is usually used in cases of small perforations that worked in a large perforation scenario and achieved adequate bone formation.

Analysis of the results of the histological study in the region of bone neoformation verified the presence of intense tissue remodeling and integration with existing tissue edge.

Some studies have shown the importance of membrane integrity only in relation to confining the graft. Careful placement of the membrane and the graft material are advocated in order to prevent extravasation. Fibrin and membranes that are rapidly resorbed and were compared in cases of sinus puncture and it was determined that rapid reabsorption membranes are not adequate for forming bone because they lose their integrity prior to bone tissue formation [13].

Furthermore, the histomorphological features of tissue proliferation indicate that the process occurred without a chronic inflammatory response [14] and, consequently, with greater mineralization. A number of studies invest in the addition of materials that promote controlled degradation, in which the biomaterial is replaced or integrated by neoformed tissue [15]. The histological results obtained herein show that both biomaterials presented partial degradation with concomitant integration of recently formed bone. Even without their presence at this stage, the presence of some phagocytic cells was observed. Despite extensive sinus rupture, the presence of blood vessels confirmed that the blood supply had been restored and was able to nourish the tissue formation.

Despite the large perforation in the sinus membrane, the biomaterials evaluated in this study and the procedures used to place them proved to be biocompatible and presented high osteogenic potential. Positioning Schneider's membrane and filling it with the graft biomaterial helped to achieve the desired osteoconduction and proliferation of bone cells in the region in which the regenerative effect of the surgical procedure was observed.
